# Comparison of a modified Sequential Organ Failure Assessment Score using RASS and FOUR

**DOI:** 10.1371/journal.pone.0229199

**Published:** 2020-02-21

**Authors:** Gabriel Piñeiro Telles, Isabella Bonifácio Brige Ferreira, Rodrigo Carvalho de Menezes, Thomas Azevedo do Carmo, Paula Lins David Pugas, Lara Freitas Marback, Maria B. Arriaga, Kiyoshi F. Fukutani, Licurgo Pamplona Neto, Sydney Agareno, Kevan M. Akrami, Nivaldo Menezes Filgueiras Filho, Bruno B. Andrade

**Affiliations:** 1 Escola Bahiana de Medicina e Saúde Pública (EBMSP), Salvador, Bahia, Brazil; 2 Universidade do Estado da Bahia (UNEB), Salvador, Bahia, Brazil; 3 União Metropolitana para o Desenvolvimento da Educação e Cultura (UNIME), Salvador, Bahia, Brazil; 4 Universidade Salvador (UNIFACS), Salvador, Bahia, Brazil; 5 Instituto Gonçalo Moniz, FIOCRUZ, Salvador, Bahia, Brazil; 6 Faculdade de Medicina da Bahia, Universidade Federal da Bahia, Salvador, Brazil; 7 Intensive Care Unit, Hospital de Cidade, Salvador, Bahia, Brazil; 8 Division of Infectious Diseases and Pulmonary Critical Care and Sleep Medicine, Department of Medicine, University of California, San Diego, California; 9 Hospital de Cidade, NEPC, Salvador, Bahia, Brazil; Wayne State University, UNITED STATES

## Abstract

**Objective:**

ICU severity scores such as the Sequential Organ Failure Assessment (SOFA) determine neurologic dysfunction based on the Glasgow Coma Scale, a tool that may be limited in a critically ill population. It remains unknown whether alternative methods to assess for neurologic dysfunction, such as FOUR and RASS, are superior. This study aimed to determine the predictive performance of a modified SOFA tool in a large Brazilian ICU cohort.

**Design:**

Prospective cohort single center study.

**Setting:**

Mixed surgical and medical ICU in Salvador, Bahia, Brazil between August 2015 and December 2018.

**Patients:**

All acutely ill ICU admissions, other than postoperative patients or those with insufficient data, were eligible for study inclusion.

**Measurements and main results:**

2147 patients were admitted to the ICU, of which 999 meeting inclusion criteria were included in the final analysis with a median age of 72 years (IQR 58–83) and a female predominance 545 (54%). The SOFA score using GCS, RASS and FOUR for the neurologic component performed marginally in the ability to predict general ICU mortality (SOFA_GCS_ AUC 0.74 vs SOFA_RASS_ AUC 0.71 and SOFA_FOUR_ AUC 0.67), with SOFA_FOUR_ performing significantly lower compared to either SOFA_RASS_ and SOFA_GCS_ (p<0.04, p<0.004 respectively). All three scores demonstrated decreased discriminate function in the mechanically ventilated population (SOFA_GCS_ AUC 0.70 vs SOFA_RASS_ AUC 0.70 and SOFA_FOUR_ AUC 0.55), though SOFA_FOUR_ remained significantly worse when compared to SOFA_GCS_ or SOFA_RASS_ (p = 0.034, p = 0.014, respectively)_._. Furthermore, performance was poor in a subset of patients with sepsis (n = 145) at time of admission (SOFA_GCS_ AUC 0.66 vs SOFA_RASS_ AUC 0.55 and SOFA_FOUR_ AUC 0.56).

**Conclusion:**

Modification of the neurologic component in the SOFA score does not appear to improve mortality prediction in the ICU.

## Introduction

The Sequential Organ Failure Assessment (SOFA) score is frequently used in the intensive care unit (ICU) to assess the incidence of organ dysfunction, guide management and aid in prognosis [1–3]. The SOFA was one of the first severity of illness scores validated for use in the ICU, initially to assess organ dysfunction in patients with sepsis [2], though recently has emerged as a tool to predict mortality in the acutely admitted ICU patient [4–6]. The score is based on major dysfunction in the following systems: hepatic, cardiovascular, neurologic, renal, respiratory and hematologic. Given the ease of calculation with data available in the daily routine of an ICU, it has become the primary method to stratify severity of illness in the ICU at time of admission [4–7].

A potential critical limitation of this tool, though, may be the assessment of neurological function based on the Glasgow Coma Scale (GCS). This scale aimed to standardize level of consciousness determinations in patients with acute brain injury [8]. However, it has expanded beyond this initial role to be used by emergency medical services and in the ICU. Although GCS is used in several ICU scoring systems, including APACHE III, SAPS III, and SOFA, it is subject to interobserver variability, and lacks verbal assessment in those undergoing mechanical ventilation [8–11]. Prior studies have attempted to modify the SOFA score, either by exclusion of the neurologic component [12,13] and even exclusion of the neurologic and hematologic component [14]. The Richmond Agitation-Sedation Scale (RASS), while designed to guide sedation in the critically ill, has emerged as an alternative tool to assess neurologic function in the ICU with minimal interobserver variability [15]. A recent study demonstrated that modification of the SOFA score by substitution of GCS for the RASS retained predictive mortality in the ICU [16].

The RASS [15,17] and the Full Outline of UnResponsiveness (FOUR) [18,19] are validated and highly reliable alternative methods to assess neurologic function in critically ill patients, irrespective of mechanical ventilation status. Given these alternative methods, we hypothesize that use of RASS and FOUR as measures of neurologic function in the SOFA score will demonstrate improved performance when compared to the GCS based SOFA score. Our ICU adopted routine collection of RASS and FOUR to evaluate neurologic function distinct from GCS. The current study aims to determine the accuracy of a modified SOFA score that replaces the neurologic GCS based component with RASS or FOUR to predict ICU mortality at time of admission of acutely ill patients.

### Study design and methodology

This was a prospective observational descriptive study in adult patients over 18 years of age, admitted to the ICU from August 2015 to December 2018, in a 22 bed ICU of an urban hospital in Salvador, Bahia, Brazil. All acutely ill patients admitted to the ICU were eligible for inclusion, while postoperative ICU admissions, those with incomplete data and transfers to other hospitals were excluded from the final analysis. Individuals with missing bilirubin data at the time were included given that levels are unlikely to be significantly altered in a non-jaundiced population.

Data on age, gender, admission source, ICU outcome and length of stay, SOFA, Charlson Comorbidity Index, use of mechanical ventilation, GCS, RASS and FOUR were prospectively recorded by the medical staff as part of routine clinical care, including patients with and without sedation use in the Epimed Monitor system, which contained all other variables of interest for this study. ICU discharge was considered end of follow-up. GCS, RASS, and FOUR values were used to determine the SOFA neurologic component score in the SOFA-NeuroGCS, SOFA-NeuroRASS and SOFA-NeuroFOUR scores as shown in Table 1. Neurologic assessment in a majority of patients undergoing mechanical ventilation was performed following intubation at time of admission to the ICU. In those individuals with RASS greater than zero (suggesting restlessness or agitation), the ICU protocol in place utilizes the CAM-ICU delirium tool to guide use of sedation. There were a limited number of patients with RASS scores greater than 0 (54 patients, 5,4%) of which 11 (1.1%) had a RASS>2. Any individual with a RASS greater than or equal to 0 (ranging from alert to combative), for the purposes of our modified score, was determined to have a SOFA neurologic point equal to 0. SOFA calculations were performed by the original method, designated as SOFA_GCS_, a RASS-based method, designated as SOFA_RASS_ and a FOUR-based method, designated as SOFA_FOUR_. SOFA_GCS,_ SOFA_RASS_ and SOFA_FOUR_ were calculated in the first 6 hours of ICU admission [2]. To test our primary hypothesis, accuracy of SOFA_GCS_, SOFA_RASS_ and SOFA_FOUR_ to predict mortality was determined by comparing the area under the curve (AUC).

**Table 1:** Neurologic SOFA score for GCS, RASS and FOUR.

SOFA Neurologic Points	GCS Score	RASS Score	FOUR Score
0	15	≥0	14–16
1	13–14	-1	11–13
2	10–12	-2	8–10
3	6–9	-3	5–7
4	3–5	-4,-5	1–4

Sequential Organ Failure Assessment (SOFA); Glasgow Coma Scale (GCS); Richmond Agitation-Sedation Scale (RASS); Full Outline of UnResponsiveness (FOUR)

Median and interquartile ranges (IQR) were used as measures of central tendency. Frequencies were compared using the Pearson’s chi-squared test. Continuous variables were compared using the Mann-Whitney U test (between two groups) or the Kruskal-Wallis test with Dunn’s multiple comparisons (between >2 groups). Correlations were tested using the Spearman’s rank correlation test.

The p-values were adjusted for multiple comparisons using the Holm-Bonferroni’s method [21]. All analyses were pre-specified. Two-sided P value < 0.05 after adjustment for multiple comparisons were considered statistically significant. To test our primary hypothesis, performance of the traditional and modified SOFA scores were compared using a two-tailed Z-test to evaluate the absolute AUC and difference in AUC derived from the empirical ROC curves produced by the NCSS Statistical Software. A Cox proportionate test analysis was performed to determine score performance when adjusted for variables that differed significantly between survivors and non-survivors to quantify risk of mortality predicted by each score. Statistical analyses were performed using SPSS 25.0 (IBM statistics), Graphpad Prism 6.0 (GraphPad Software, San Diego, CA) and JMP 12.0 (SAS, Cary, NC, USA). Ethics approval and waiver of consent to participate was approved by the Research Ethics Committee of Hospital Ana Nery under the number 2.571.265 and CAAE 52892315.1.0000.0045.

## Results

**Fig 1:**
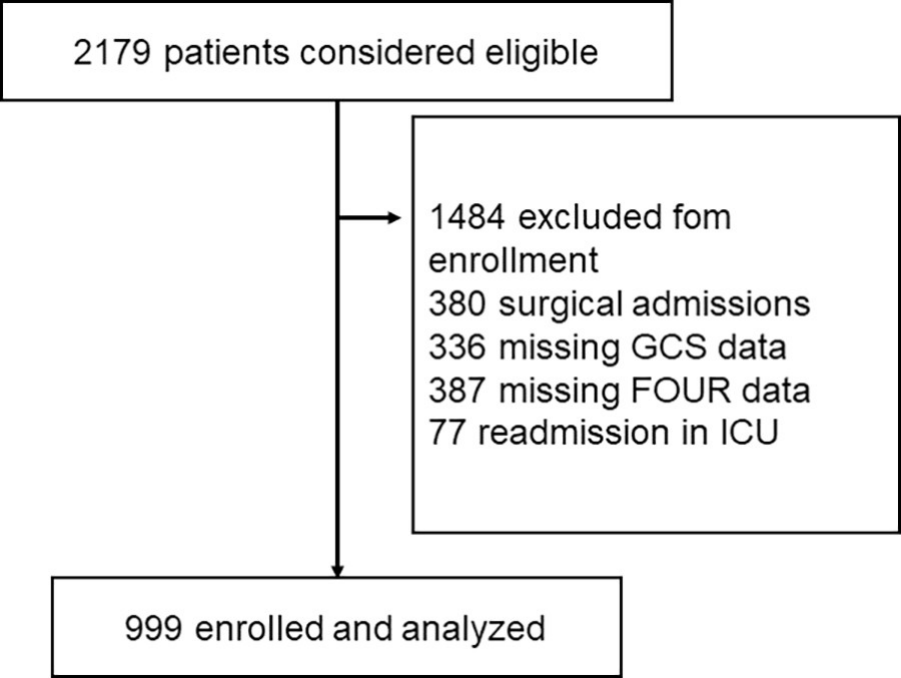
Flowchart of study participants.

Over the course of the study period, 2179 patients were admitted to the ICU with 1180 excluded from final analysis for the following reasons: 380 elective post-operative admissions, 336 missing GCS data, 387 missing FOUR data and 77 who were readmitted to the ICU. The final sample consisted of 999 patients (Fig 1).

The median age of the included cohort was 72 years old (IQR 57–83) with a female predominance (n = 544, 54.4%) (Table 2). Individuals requiring mechanical ventilation constituted a minority of patients (n = 85, 8.5%). The SOFA modified for RASS and FOUR similarly under-predicted mortality in the intubated population. Moreover, non-survivors were more likely to require vasopressors (n = 17, 14.4%) compared with those who survived (n = 13, 1.4%) for p<0.001. Neurologic scores including GCS, RASS and FOUR were decreased significantly in non-survivors compared to survivors (median n = 14, 9–15 vs n = 15, 15–15, p< 0.001). Mortality was significantly increased in those with an infectious or renal indication for ICU admission.

**Table 2:** Study population characteristics by mortality outcome.

Population Characteristics	All encounters (n = 999)	Non-survivors (n = 118)	Survivors (n = 883)	p-value
Age (years, median, IQR)	72 [57–83]	82 [69.5–89.5]	71 [56–81]	< 0.001^a^
Female sex (n, %)	545 (54.5)	57 (43.2)	488 (55.2)	0.128
ICU Length of Stay (Days)	4 [3–7]	9 [3–17.5]	5 [3–7]	< 0.001^a^
Use of Vasopressors (n, %)	34 (3.4)	17 (14.4)	13 (1.4)	< 0.001^a^
Use of Mechanical Ventilation (n, %)	86 (8.5)	41 (34.7)	45 (5)	< 0.001^a^
Neurologic Assessment Scores				
FOUR	16 [16–16]	14 [11–16]	16 [16–16]	< 0.001^a^
Glasgow	15 [15–15]	14 [9–15]	15 [15–15]	< 0.001^a^
RASS	0 [0–0]	0 [–3–0]	0 [0–0]	< 0.001^a^
ICU Indications (n, %)				
Cardiologic	244 (24.4)	7 (5.9)	237 (26.8)	< 0.001
Pulmonary	61 (6.1)	11 (9.3)	50 (5.6)	0.15
Infection	192 (19.2)	39 (3.3)	153 (17.3)	0.002
Renal	42 (4.2)	10 (8.4)	32 (3.6)	0.023
Others	460 (46)	50 (42.3)	410 (46.4)	< 0.001

Values shown in median and IQR

Sequential Organ Failure Assessment (SOFA); Glasgow Coma Scale (GCS); Richmond Agitation-Sedation Scale (RASS); Full Outline of UnResponsiveness (FOUR).

^a^Kruskal–Wallis

**Fig 2:**
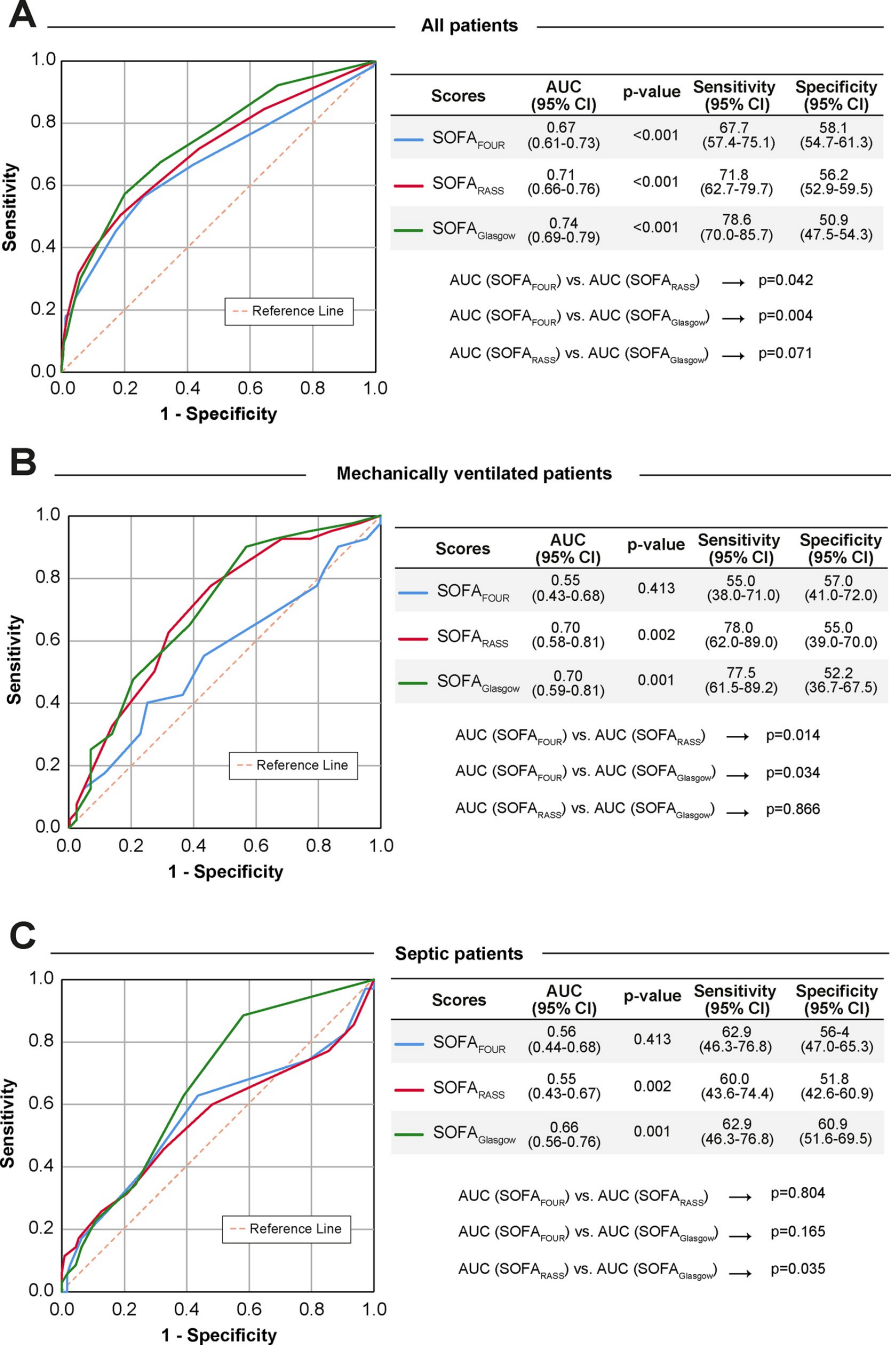
Comparison of receiver operating characteristic (ROC) curves for prediction of ICU mortality by Sequential Organ Failure Assessment (SOFA) using Glasgow Coma Scale (GCS), Richmond Agitation and Sedation Scale (RASS) and Full Outline of UnResponsiveness (FOUR) neurologic assessment substitutions in the total cohort (A), subset undergoing mechanical ventilation (B) and septic subset (C). Comparisons between the absolute and differences of AUC were considered significant for p<0.05. AUC = area under the curve.

**Fig 3:**
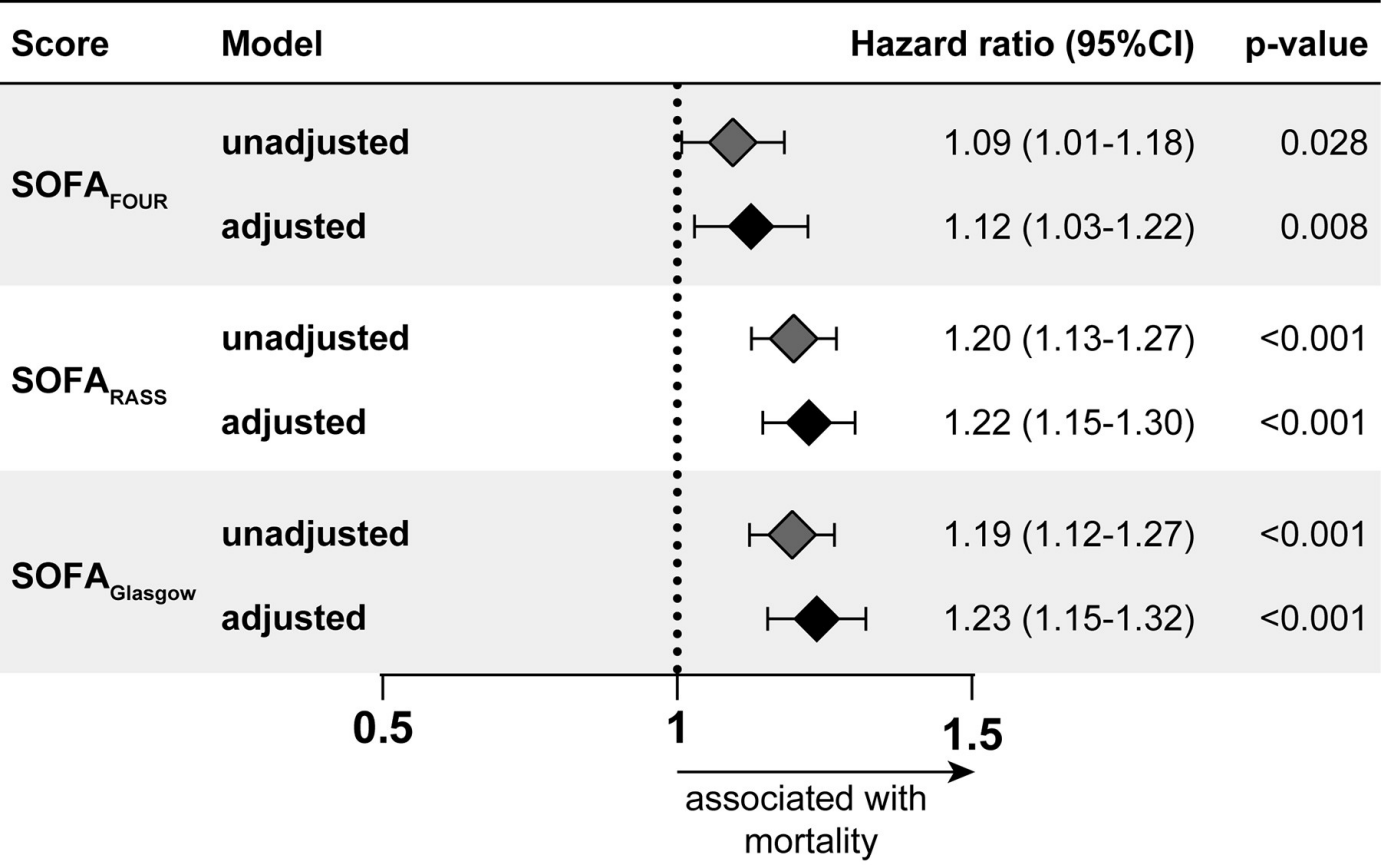
Adjusted and unadjusted Cox regression model for ICU mortality. The effects of traditional and modified SOFA upon survival are constant over time and did not vary when each one was adjusted for age, gender and BMI (body mass index).

When discriminate performance was assessed, accuracy of the modified SOFA with RASS or FOUR was comparable to SOFA_GCS_. However, the SOFA_FOUR_ demonstrated significantly decreased discriminate function (AUC 0.67) for the overall cohort compared to both SOFA_GCS_ (AUC 0.74) and SOFA_RASS_ (AUC 0.71)), p = 0.042 and p = 0.004, respectively as seen in Fig 2A. There was no significant difference in performance between GCS and RASS based SOFA for the overall cohort. All three scores underperformed when evaluated in patients undergoing mechanical ventilation, a population for whom FOUR and RASS were predicted to demonstrate improved performance (Fig 2B). Specifically, SOFA_FOUR_ (AUC 0.55) performed significantly worse when compared to GCS (AUC 0.70) and RASS (0.70), p = 0.014 and p = 0.034 respectively, while no significant difference was detected between SOFA based on RASS and GCS. The modified and original SOFA scores continued to perform poorly when evaluated in those admitted with sepsis (Fig 2C), though the RASS based SOFA was significantly worse compared to the GCS based SOFA (AUC 0.55 vs 0.66, p = 0.035). Cox proportionate testing confirmed findings in the ROC analysis with all scores marginally predicting mortality, even when adjusting for significant covariates in survivors and non-survivors (Fig 3).

## Discussion

This is one of the first prospective cohort studies in the ICU to evaluate whether substitution of the GCS in the SOFA score with RASS and FOUR improved prediction of ICU mortality at the time of admission. In contrast to our initial hypothesis, our findings suggest that substitution with FOUR is inferior to both the RASS and GCS based SOFA, even in a mechanically ventilated population. In contrast, it appears that RASS substitution for GCS may be a more convenient measure of neurologic function with marginal loss of SOFA score performance. Why these alternative methods did not surpass GCS in measurement of neurologic dysfunction is unclear. The reduced performance of the FOUR modified score in particular, compared to either RASS or GCS, suggests that patient populations distinct from a neurocritical cohort in whom this scale was developed may limit the generalizability of the FOUR [18, 19]. It may be that in the absence of severe neurologic dysfunction, the discriminate function of the FOUR score may be inferior to the RASS or GCS, as was the case in our population with primarily cardiac, pulmonary or infectious causes of ICU admission.

The Cox proportionate analysis adjusted for baseline characteristics (including age and comorbidities from the Charlson and Modified Frailty index) demonstrated marginal differences in performance of the modified and the original scores, contrary to our initial hypothesis. There may be additional unmeasured local factors responsible for ICU mortality that explain why the SOFA severity score performs poorly independent of modification of the neurologic component. Our data suggest that only RASS modified SOFA is a possible alternative to SOFA_GCS_ for prediction of mortality in the first 24 hours in the ICU. Our findings demonstrate similar performance of the RASS modified and original SOFA scores in the general ICU population, suggesting that alternative neurologic scale for SOFA with RASS may be effective in sedated patients and those undergoing mechanical ventilation [15,16].

Despite our study’s strength to support the use of alternatives measures of neurologic function in SOFA, we acknowledge a number of limitations. First, as a single center study there may be unknown confounding factors bias. However, given the significant size of our cohort and similar severity of illness in the general ICU population to other studies, it is unlikely that heterogeneity influenced the results found here. Second, the limited number of participants undergoing mechanical ventilation may have impacted the poor performance of all three scores in the intubated population. Discriminate function may improve in cohorts with increased use of mechanical ventilation, though, our findings highlight the poor performance of SOFA with and without modification. The lack of discriminate performance of all 3 SOFA scores in our population may be a consequence of a primarily elderly cohort whose mortality is not accurately predicted by these scores with unknown patient factors impacting score performance. Further studies are needed to clarify these findings. While 30-day mortality and morbidity following ICU discharge was not available for our cohort, our study focused on the severity of illness at time of ICU admission of an acutely ill cohort with the primary outcome of inpatient ICU mortality. Finally, despite recent studies finding improved predictive ability of SOFA in those with sepsis, our results demonstrated poor performance for both the original and modified SOFA scores, suggesting possible local patient or methodological factors in the sepsis subset [20,21].

## Conclusion

Despite routine use in various medical environments, GCS may not be the most effective neurological scale for the ICU. While RASS and FOUR are convenient, our findings only support RASS substitution as a reasonable alternative for the neurologic component in the SOFA score. Further studies are required to determine whether these modifications may demonstrate improved accuracy in ICU mortality prediction in specific subpopulations, including those who are post-operative.

## Supporting information

S1 File (XLSX)

## References

[1] Knaus WA, Draper EA, Wagner DP, Zimmerman JE. APACHE II: a severity of disease classification system. Crit Care Med. 1985 10;13(10):818–29. 3928249

[2] Vincent JL, Moreno R, Takala J, Willatts S, De Mendonça A, Bruining H, et al The SOFA (Sepsis-related Organ Failure Assessment) score to describe organ dysfunction/failure. On behalf of the Working Group on Sepsis-Related Problems of the European Society of Intensive Care Medicine. Intensive Care Med. 1996 7;22(7):707–10. 10.1007/bf01709751 8844239

[3] Marshall JC, Cook DJ, Christou N V, Bernard GR, Sprung CL, Sibbald WJ. Multiple organ dysfunction score: a reliable descriptor of a complex clinical outcome. Crit Care Med. 1995 10;23(10):1638–52. 10.1097/00003246-199510000-00007 7587228

[4] Vincent JL, de Mendonça A, Cantraine F, Moreno R, Takala J, Suter PM, et al Use of the SOFA score to assess the incidence of organ dysfunction/failure in intensive care units: results of a multicenter, prospective study. Working group on &quot;sepsis-related problems&quot; of the European Society of Intensive Care Medicine. Crit Care Med. 1998 11;26(11):1793–800. 10.1097/00003246-199811000-00016 9824069

[5] Moreno R, Vincent JL, Matos R, Mendonça A, Cantraine F, Thijs L, et al The use of maximum SOFA score to quantify organ dysfunction/failure in intensive care. Results of a prospective, multicentre study. Working Group on Sepsis related Problems of the ESICM. Intensive Care Med. 1999 7;25(7):686–96. 10.1007/s001340050931 10470572

[6] Ferreira FL, Bota DP, Bross A, Mélot C, Vincent JL. Serial evaluation of the SOFA score to predict outcome in critically ill patients. JAMA. 2001 10 10;286(14):1754–8. 10.1001/jama.286.14.1754 11594901

[7] Badreldin A, Elsobky S, Lehmann T, Brehm B, Doenst T, Hekmat K. Daily-Mean-SOFA, a New Derivative to Increase Accuracy of Mortality Prediction in Cardiac Surgical Intensive Care Units. Thorac Cardiovasc Surg. 2012 2 3;60(01):043–50. 10.1055/s-0031-1295568 22215501

[8] Rowley G, Fielding K. Reliability and accuracy of the Glasgow Coma Scale with experienced and inexperienced users. Lancet Lond Engl. 1991 3 2;337(8740):535–8. 10.1016/0140-6736(91)91309-i 1671900

[9] Liddy M. Chen CMMTLMWJS. Interobserver variability in data collection of the Apache II score in teaching and community hospitals. Crit Care Med. 1999 9 1;27(9):1999–2004. 10.1097/00003246-199909000-00046 10507631

[10] Teasdale G, Jennett B. Assessment of coma and impaired consciousness. A practical scale. Lancet Lond Engl. 1974 7 13;2(7872):81–4. 10.1016/s0140-6736(74)91639-0 4136544

[11] Kho ME, McDonald E, Stratford PW, Cook DJ. Interrater reliability of APACHE II scores for medical-surgical intensive care patients: a prospective blinded study. Am J Crit Care Off Publ Am Assoc Crit-Care Nurses. 2007 7;16(4):378–83. 17595370

[12] Junger A, Engel J, Benson M, Böttger S, Grabow C, Hartmann B, et al Discriminative power on mortality of a modified Sequential Organ Failure Assessment score for complete automatic computation in an operative intensive care unit. Crit Care Med. 2002 2;30(2):338–42. 10.1097/00003246-200202000-00012 11889305

[13] Nates JL, Cárdenas-Turanzas M, Ensor J, Wakefield C, Wallace SK, Price KJ. Cross-validation of a modified score to predict mortality in cancer patients admitted to the intensive care unit. J Crit Care. 2011 8;26(4):388–94. 10.1016/j.jcrc.2010.10.016 21195582

[14] Demandt AMP, Geerse DA, Janssen BJP, Winkens B, Schouten HC, van Mook WNKA. The prognostic value of a trend in modified SOFA score for patients with hematological malignancies in the intensive care unit. Eur J Haematol. 2017 10;99(4):315–22. 10.1111/ejh.12919 28656589

[15] Sessler CN, Gosnell MS, Grap MJ, Brophy GM, O’Neal P V., Keane KA, et al The Richmond Agitation–Sedation Scale. Am J Respir Crit Care Med. 2002 11 15;166(10):1338–44. 10.1164/rccm.2107138 12421743

[16] Vasilevskis EE, Pandharipande PP, Graves AJ, Shintani A, Tsuruta R, Ely EW, et al Validity of a Modified Sequential Organ Failure Assessment Score Using the Richmond Agitation-Sedation Scale. Crit Care Med. 2016 Jan;44(1):138–46. 10.1097/CCM.0000000000001375 26457749 PMC4748963

[17] Ely EW, Truman B, Shintani A, Thomason JWW, Wheeler AP, Gordon S, et al Monitoring Sedation Status Over Time in ICU Patients. JAMA. 2003 6 11;289(22):2983 10.1001/jama.289.22.2983 12799407

[18] Iyer VN, Mandrekar JN, Danielson RD, Zubkov AY, Elmer JL, Wijdicks EFM. Validity of the FOUR score coma scale in the medical intensive care unit. Mayo Clin Proc. 2009 8;84(8):694–701. 10.1016/S0025-6196(11)60519-3 19648386 PMC2719522

[19] Jamal A, Sankhyan N, Jayashree M, Singhi S, Singhi P. Full Outline of Unresponsiveness score and the Glasgow Coma Scale in prediction of pediatric coma. World J Emerg Med. 2017;8(1):55–60. 10.5847/wjem.j.1920-8642.2017.01.010 28123622 PMC5263038

[20] Seymour CW, Liu VX, Iwashyna TJ, et al Assessment of Clinical Criteria for Sepsis: For the Third International Consensus Definitions for Sepsis and Septic Shock (Sepsis-3). JAMA. 2016;315(8):762–774. 10.1001/jama.2016.0288 26903335 PMC5433435

[21] Kaukonen KM, Bailey M, Suzuki S, Pilcher D, Bellomo R. Mortality related to severe sepsis and septic shock among critically ill patients in Australia and New Zealand, 2000–2012. JAMA. 2014;311(13):1308–1316. 10.1001/jama.2014.2637 24638143

